# Students working against tobacco: A novel educational program to improve Canadian medical students’ tobacco counselling skills

**Published:** 2018-05-31

**Authors:** Deanna Lammers, Zach Zhang, Iuliia Povieriena, Andrew Pipe

**Affiliations:** 1 Faculty of Medicine, University of Ottawa, Ontario, Canada; 2 Division of Prevention and Rehabilitation, University of Ottawa Heart Institute, Ontario, Canada

## Abstract

**Background:**

Medical professionals should be appropriately trained in the field of smoking cessation counseling and be familiar with related tobacco-control issues. Sadly, Canadian medical students receive little education regarding smoking cessation.

**Methods:**

University of Ottawa medical students created Students Working Against Tobacco (SWAT), a program that provides its members with tobacco education and opportunities to discuss tobacco use, smoking prevention and cessation with elementary-school students. Surveys assessing student knowledge and confidence in addressing tobacco issues were administered to the participating students at the start of the program and following their delivery of a school presentation.

**Results:**

Students initially lacked knowledge, skills and experience in addressing tobacco issues and discussing smoking prevention and cessation counselling. Following their involvement in the SWAT program, students’ smoking cessation counselling knowledge and skills improved, and they expressed confidence in becoming more engaged in this important preventive health issue.

**Conclusion:**

Until smoking cessation is incorporated into undergraduate medical education programs, gaps will remain in the preparation of tomorrow’s physicians regarding the provision of effective smoking cessation counselling and their broader understanding of this important health issue. Currently, there are constraints limiting the number of medical undergraduates that SWAT is able to involve and influence.

## Introduction

Smoking is the leading cause of preventable disease, disability and death worldwide. If smoking rates do not significantly decline, almost 1 billion deaths secondary to tobacco use will have occurred by the end of the 21^st^ century.^1^

Currently 18% of Canadians smoke,^2,3^ with the majority beginning in their youth.^4,5^ Among adolescents, 24% have tried a cigarette, 28% have tried a tobacco product and 4% are current smokers.^5^ There is no national Canadian data describing the use of e-cigarettes by youth, but 10% of grade 9 students in a representative cohort have tried electronic cigarettes.^6^

A large number of smokers attempt cessation each year, but only 5% are successful.^7^ With the provision of brief advice, healthcare workers can have a positive impact on smoking cessation;^8-10^ with specific assistance and appropriate support, physicians can obtain higher cessation rates.^11^ It is important, therefore, that physicians are appropriately trained to promote smoking cessation.^4,9,10,12,13^

Unfortunately, Canadian medical students receive little education regarding smoking cessation.^9,14,15^ An assessment of 11 of the 17 Canadian medical schools revealed that 64% of those schools provide less than three hours of smoking cessation education, 64% do not examine their students on smoking cessation and 73% do not provide an opportunity to rehearse cessation counselling skills.^14^ This lack of training is reflected in the attitudes of graduating medical students from one university: only half of them felt capable of counselling and treating patients for smoking cessation. Surprisingly, one-third did not view smoking cessation as being a healthcare priority.^15^ Multiple studies have demonstrated the positive association between decreased physician knowledge about tobacco cessation and likelihood of addressing the smoking behaviour of their patients.^4,10,12,16^

At the time of this study, the University of Ottawa, medical students received only a two-hour lecture on smoking and tobacco products as part of a course addressing psycho-social issues in medicine. To address this shortcoming, medical students created an interest group: Students Working Against Tobacco (SWAT). The goals of the organization are to improve future physicians’ tobacco education and counselling skills while familiarizing them with the importance of addressing Canada’s leading cause of preventable disease, disability and death.

Herein, we describe the possible effect of an extracurricular anti-tobacco advocacy and education program on the knowledge of medical students regarding tobacco and health, their comfort in addressing smoking cessation, and the acquisition of counselling skills.

## Methods

### Learning opportunities for medical students through SWAT

During the academic year, SWAT student-leaders organize frequent, relevant, presentations by health care professionals familiar with tobacco control and smoking cessation issues. Topics include: nicotine addiction; the nature of tobacco products including e-cigarettes and shisha; socio-economic correlates of smoking behaviour; co-existing substance use; patient perspectives on cessation; cessation techniques and programs; smoking cessation advocacy; and, community cessation resources. Each talk is delivered during a lunch hour period and provides students with knowledge not currently provided in any part of the University of Ottawa medical school curriculum. In the 2015-16 year, 140 students joined SWAT and an average of 19 students attended each of the seven talks. Membership in SWAT was similar to that of most other special interest groups in the University of Ottawa Faculty of Medicine.

An important component of the program is the opportunity for medical students to deliver tobacco presentations in local elementary schools. Each year, SWAT leaders contact Ottawa schools, offering the presentations to supplement the schools’ health curriculum. Additionally, SWAT leaders prepare and provide an annotated slide set and a mandatory training session to SWAT members prior to school visits. The training session includes detailed explanations of the presentation material as well as tips on engaging children. The presentation addresses the design and composition of a cigarette, the health impacts of smoking, the financial costs of tobacco addiction, e-cigarettes, the stigma and psychological impact of smoking behaviour, and the marketing strategies used by tobacco companies.

### Evaluation of the SWAT program

In the 2015-2016 school year, SWAT members completed a self-assessment survey at the start of their training session and, ultimately, following the completion of a school presentation. The initial survey evaluated the students’ baseline tobacco cessation knowledge and counselling skills. The survey was repeated to evaluate any subsequent changes in knowledge and skills. The primary objective was to assess the SWAT program’s effectiveness in developing medical students’ clinical counselling and teaching skills and their comfort level in addressing smoking cessation and prevention. The survey also assessed students’ attitudes regarding the role of health care professionals in assisting with cessation.

The survey consisted of 24 questions and was based on questionnaires used in recent studies examining health care professionals’ tobacco use and cessation practices.^17-19^ The size of our sample did not permit inferential statistical analysis; our results are descriptive only. No incentive was offered for completing the survey.

## Results

In the past 5 years, 53 pre-clerkship medical students have given presentations to 38 classes of children in grades 4-10, in both French and English. During the 2015-16 academic year, 13 pre-clerkship medical students underwent training; six medical students delivered four presentations.

### Participants

The characteristics of the participating medical students are displayed in Table 1. Only one student had ever smoked or was smoking at the time. All six students who completed a school presentation were female and in their second year of medical school. Five of the six students completed the post-presentation survey.

**Table 1:** Demographic characteristics of SWAT training session participants

	Number/Total
Gender	
Male	4/13
Female	9/13
Medical School Year	
Year 1	4/13
Year 2	9/13
Smoker (Current or Ever)	1/13

### Students’ Baseline

**Figure 1:**
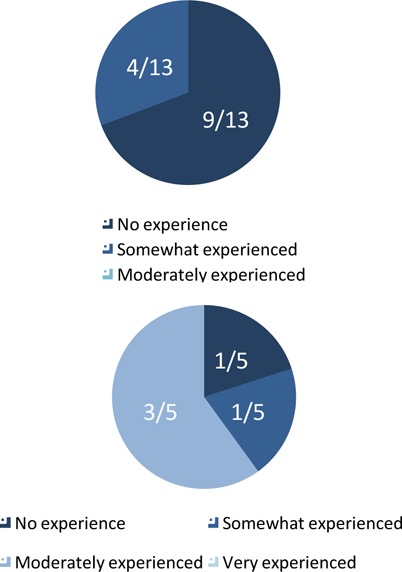
University of Ottawa SWAT members’ pre-training (top) and post-presentation (bottom) amount of experience with public tobacco education

**Figure 2:**
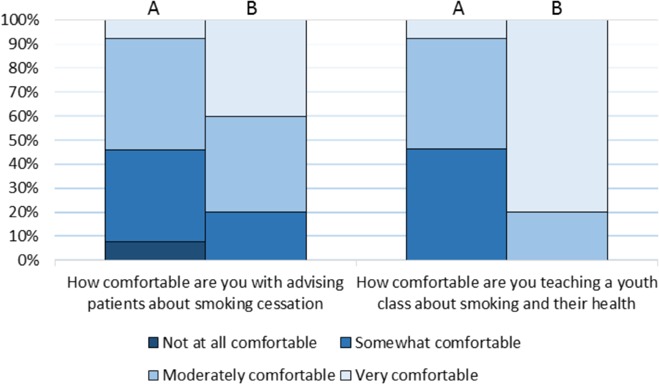
University of Ottawa medical students’ pre-training (A) and post-presentation (B) comfort level with discussing smoking cessation

Prior to training, all 13 SWAT members stated they had either no experience (4/13) or were somewhat experienced (9/13) in educating others about tobacco (Figure 1). Almost half were not at all comfortable or somewhat comfortable advising patients about smoking cessation and teaching youth about tobacco (6/13, Figure 2).

**Figure 3:**
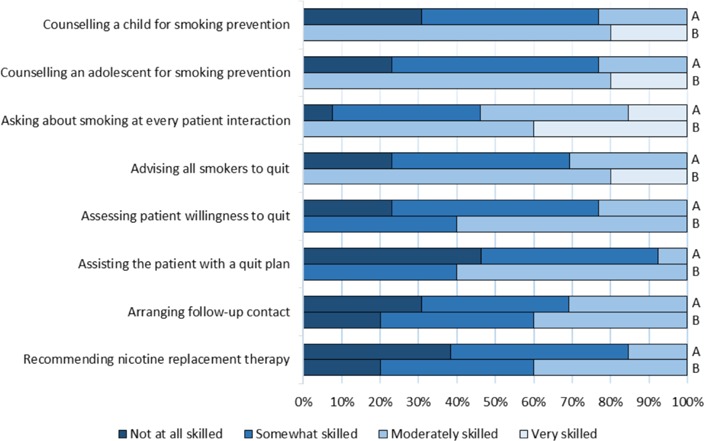
University of Ottawa medical students’ pre-training (A) and post-presentation (B) smoking cessation and prevention skills

Students were asked about their skill set regarding smoking cessation counselling. The results are summarized in Figure 3. The majority of students reported having no skills or being only somewhat skilled in: counselling a child or adolescent regarding smoking prevention (10/13); asking about smoking at every patient interaction (6/13); advising all smokers to quit (9/13); assessing patient willingness to quit (10/13); assisting a patient with a quit plan (12/13); arranging follow-up (9/13); and, recommending nicotine replacement therapy (11/13).

**Figure 4:**
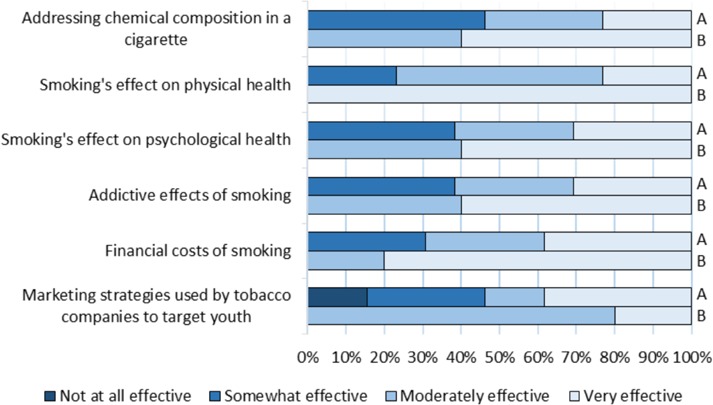
University of Ottawa medical students’ pre-training (A) and post-presentation (B) opinion on effective strategies for youth tobacco prevention and cessation

A summary of the perceived effectiveness of commonly used approaches to youth tobacco prevention is provided in Figure 4. All students considered the approaches as somewhat, moderately or very effective, with the exception of two students who believed discussing tobacco company marketing strategies as not at all effective.

**Figure 5:**
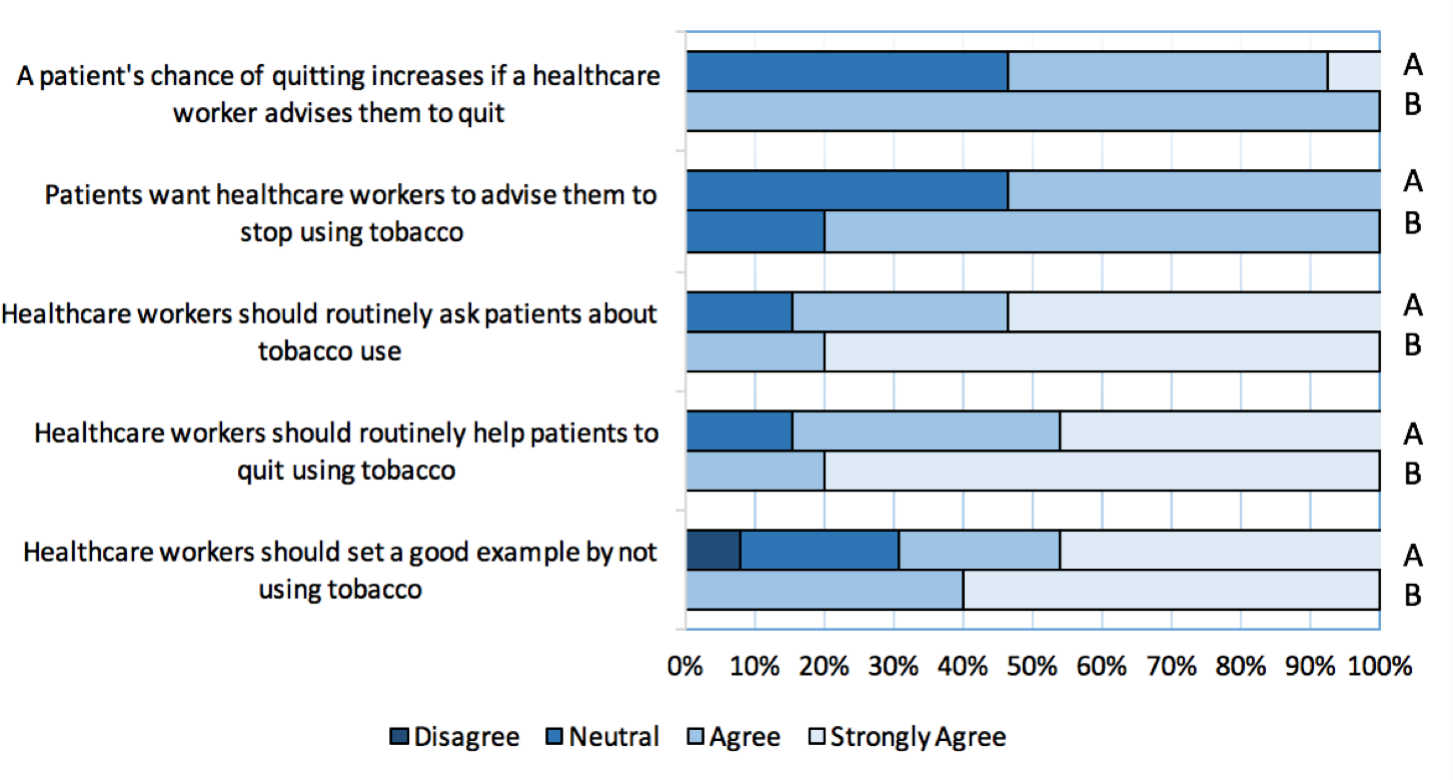
University of Ottawa medical students’ pre-training (A) and post-presentation (B) attitudes on patient smoking cessation

When asked whether they agreed with various belief statements regarding the role of the health care provider in patient smoking cessation, many students reported that they disagreed or felt neutral that healthcare workers: can increase a patient’s chance of quitting (6/13); should routinely ask patients about tobacco use (2/13); should routinely help patients to quit using tobacco (2/13); and, should set a good example by not using tobacco (4/13) (Figure 5).

### Students’ improvement

Following the completion of a school presentation, students’ comfort and skills regarding smoking cessation improved. They felt moderately experienced in public tobacco education (3/5), very comfortable teaching youth about the topic (4/5) and very comfortable advising patients (2/5) (Figures 1, 2). Students reported substantial improvement in terms of their smoking cessation and prevention counselling abilities (Figure 3). All students agreed the commonly used tobacco prevention and cessation strategies were moderately or very effective (Figure 4).

A change in the student perception of the role of a physician in smoking cessation also occurred; all students agreed or strongly agreed that a healthcare worker plays an important role in the identification, assessment, and treatment of smokers. One student remained neutral on whether patients want healthcare providers to recommend quitting (Figure 5).

## Discussion

It has already been noted that a significant proportion of graduating Canadian medical students from one university felt they had not received enough education in smoking cessation.^15^ Although there were few participants, our results may indicate University of Ottawa pre-clerkship medical students may lack knowledge, skills, and experience in smoking cessation counselling. Astonishingly, many were skeptical about the role of a physician in helping patients to quit despite abundant evidence that suggests otherwise.^8-11^ Furthermore, students were unaware of evidence-based approaches to smoking prevention, with some skeptical that industry denormalization is influential, when in fact, it has demonstrated significant effectiveness in addressing and preventing tobacco use among youth.^20^

SWAT was originally created to increase student exposure and education in tobacco cessation. The development of SWAT has augmented learning and led to greater engagement in addressing this important healthcare issue. Self-assessments were the fundamental tool employed in our study. They have been identified as a reliable method in evaluating competence and are seen as possessing utility in the assessment of educational programs.^21^ Unfortunately, the small number of participants in the cohort study negated the possibility of statistical analysis to more accurately demonstrate the impact of the SWAT program. Further and more rigorous research is needed.

### Conclusion

The importance of smoking cessation training should be reflected in the Canadian medical schools’ curricula. Sadly, the dangers of tobacco and the approaches to cessation appear to be unacknowledged in medical education. That such matters are seemingly absent is ironic given the human and financial costs incurred as a direct consequence of tobacco addiction. Smokers lose a decade of life expectancy from nicotine addiction and smoking cessation is the most important of all preventive initiatives.^22^

Building on their experience, SWAT leaders at the University of Ottawa have worked with leading Canadian experts to create an evidence-based clinical skills development tutorial on smoking cessation for medical students. A trial of this initiative was recently concluded. It is hoped that it will be incorporated into the medical curriculum in the near future. The magnitude of the global tobacco epidemic and the stated desire to reduce smoking in Canada to “less than 5% by 2035” require that tomorrow’s medical practitioners are able to assist with smoking cessation.^23^
